# The impact of COVID-19 vaccination on patients with congenital heart disease in England: a case-control study

**DOI:** 10.1136/heartjnl-2024-324470

**Published:** 2024-10-11

**Authors:** Catriona Harrison, Simon Frain, Farideh Jalalinajafabadi, Simon G Williams, Bernard Keavney

**Affiliations:** 1 Division of Cardiovascular Sciences, School of Medical Sciences, Faculty of Biology, Medicine and Health, Manchester Academic Health Science Centre, The University of Manchester, Manchester, UK; 2 Manchester Heart Institute, Manchester University NHS Foundation Trust, Manchester, UK

**Keywords:** Cohort Studies, COVID-19, Electronic Health Records, Heart Defects, Congenital

## Abstract

**Background:**

Studies predating widespread COVID-19 vaccination identified patients with congenital heart disease (CHD) as a group at increased risk of severe outcomes from COVID-19. Here we evaluate the impact of vaccination on COVID-19 outcomes among patients with CHD.

**Methods:**

We conducted a case-control study using linked English electronic health records (n=3 18 135). Patients with CHD were matched with controls by age, sex, ethnicity and GP practice. The ‘prevaccination’ cohort comprised unvaccinated patients with CHD and matched controls with first-recorded SARS-CoV-2 infection between 1 March and 8 December 2020 (7805 cases, 27 620 controls). The ‘post-vaccination’ cohort comprised vaccinated patients with CHD and matched controls with first-recorded SARS-CoV-2 infection between 1 March 2021 and 1 April 2022, at least 14 days after vaccination (57 550 cases, 225 160 controls). Odds of severe COVID-19 outcomes were compared using conditional logistic regression. We also compared the rate at which vaccine efficacy diminished, and the incidence of vaccine-associated complications.

**Results:**

Compared with the prevaccination cohort, postvaccination patients with CHD exhibited markedly reduced rates of COVID-19-related hospitalisation (0.5% vs 15.8%) and mortality rates (0.5% vs 4.6%). Compared with vaccinated controls, vaccinated patients with CHD remained at increased risk of hospitalisation (0.5% vs 0.2%, adjusted OR 2.24 (1.88–2.65); p<0.001) and death (0.5% vs 0.3%, adjusted OR 1.81 (1.54–2.13); p<0.001). There was no evidence that vaccine efficacy declined faster in patients with CHD, or that patients with CHD experienced a larger increase in incidence of myocarditis, pericarditis or thrombotic events.

**Conclusion:**

We observed a lower absolute risk of hospitalisation and death from COVID-19 in CHD patients after vaccination. However, in vaccinated CHD patients, an elevated risk of severe outcomes persists compared with vaccinated people without CHD. These results emphasise the importance of vaccination in the CHD population, and of vigilance among care providers dealing with COVID-19 infection in CHD patients, even if fully vaccinated.

**WHAT IS ALREADY KNOWN ON THIS TOPIC**

- Studies have shown that patients with congenital heart disease (CHD) carry an excess risk of hospitalisation and death following COVID-19 infection.
- These previous studies predate large-scale vaccination programmes and were therefore unable to determine the impact of vaccination on these excess risks.

**WHAT THIS STUDY ADDS**

- Here we use electronic health records from 318 000 people in England to determine the impact of vaccination in patients with CHD, indicating that vaccination is associated with a marked reduction in the absolute risk of death and hospitalisation in patients with CHD with COVID-19.
- However, although these risks were significantly reduced, when compared with the matched control group, we found that vaccinated patients with CHD still carry additional COVID-19 risk; more than twice the odds of hospitalisation and 81% higher odds of death.
- Additionally, we assess the incidence of rare complications associated with COVID-19 vaccination in patients with CHD, finding no evidence of excess risk relative to baseline compared with patients without CHD.

**HOW THIS STUDY MIGHT AFFECT RESEARCH, PRACTICE OR POLICY**

- These findings emphasise the importance of vaccination in the CHD population and suggest increased vigilance on the part of care providers when dealing with COVID-19 in vaccinated patients with CHD remains appropriate.
- Additionally, despite a pre-existing higher risk of cardiac inflammation and thrombotic events in the CHD population, the increase in risk experienced by patients with CHD after vaccination is proportional to that observed in vaccinated people without CHD.

## Introduction

 The COVID-19 pandemic brought unprecedented challenges to healthcare systems, revealing the complex interplay between viral infections and pre-existing health conditions.^1 2^ Among vulnerable populations identified during the pandemic, individuals with congenital heart disease (CHD) emerged as a cohort with heightened susceptibility to severe outcomes following COVID-19 infection.^3^ Early studies, including our own, underscored the elevated risk of hospitalisation and mortality in CHD patients,^3^,^7^ highlighting the clinical need for targeted interventions to mitigate these risks.

CHD encompasses a diverse array of structural heart abnormalities present at birth, of widely differing severities. The interplay of cardiac anomalies, varying degrees of physiological compromise including abnormal lung function,^8^ and the potential for associated comorbidities,^9 10^ renders patients with CHD particularly susceptible to the adverse outcomes of respiratory infections such as COVID-19. COVID-19 vaccination is the most significant public health intervention available for this disease, and it has been widely taken up by the population of the UK. Previous studies of CHD and COVID-19 used data predating large-scale vaccination programmes and were therefore unable to determine the impact of vaccination on the excess risks associated with COVID-19 in patients with CHD. In addition, the incidence of rare complications associated with COVID-19, such as myo/pericarditis^11^ and venous thromboembolic disease,^12^,^16^ has not been assessed in the CHD patient population in the context of vaccination. Here we use comprehensive electronic health data from 318 135 people resident in England to quantify the excess risks of severe COVID-19 outcomes, and the incidence of vaccine-associated complications, in vaccinated patients with CHD compared with vaccinated people without CHD.

## Methods

### Data sources and study design

English electronic health records (n=64 576 431) were accessed through the National Health Service Secure Data Environment service for England, via the British Heart Foundation Data Science Centre’s CVD-COVID-UK/COVID-IMPACT Consortium and linked as described in online supplemental figure 1. Baseline characteristics, diagnoses and procedures were extracted from the General Practice Extraction Service Data for Pandemic Planning and Research (GDPPR), Hospital Episode Statistics Outpatient (HES-OP) and Hospital Episode Statistics Admitted Patient Care (HES-APC) data.

Data were extracted representing two time periods: prewidespread vaccination and postwidespread vaccination. For each of these ‘windows’, a cohort of patients with CHD and matched controls was selected. The prevaccination window was defined as the period from 1 March 2020 to 8 December 2020, the date of the first UK COVID-19 vaccination.^17^ The postvaccination window encompassed the time between 1 March 2021 and 1 April 2022. The second assessment window was chosen based on the period from the successful completion of vaccination of the top four priority groups^18^ to the end of the National Health Service contact tracing service and scale-down of testing capacity.^19^

Eligibility for the study required both patients with CHD and their matched controls to satisfy four criteria:^1^ being alive at the beginning of the study window,^2^ residing in England,^3^ having at least one record in the general practice data set, and^4^ contracting SARS-CoV-2 for the first time during the relevant study window. Patients in the postvaccination cohort also had to have received at least one COVID-19 vaccination at least 14 days prior to their first SARS-CoV-2 infection.

### Identification of CHD cases

Primary care, hospital admission and outpatient records (all diagnostic positions) from eligible patients were filtered for clinical codes indicating CHD diagnosis or procedure. Patients of any age, with CHD-specific codes (online supplemental table 1A) were classified as cases (detailed in online supplemental methods). See online supplemental table 2 for CHD subtypes. To investigate the effect of CHD severity on COVID-19 outcomes, cases were assigned a severity classification (mild/moderate/severe) based on European Society of Cardiology guidelines.^20^

### COVID-19 infection and vaccination

Records of SARS-CoV-2 infection and COVID-19 vaccination were linked to each patient. The earliest SARS-CoV-2 infection for each patient was identified by the presence of specific Systemized Nomenclature of Medicine (SNOMED), International Classification of Diseases (ICD-10) or Read codes (online supplemental methods). Patients who had not contracted SARS-CoV-2 for the first time during the study window were excluded from the cohort.

For the postvaccination time window, the vaccination status of each person on their first SARS-CoV-2 infection was identified (online supplemental methods). Vaccination status was based on the number of vaccinations they had received 14 days prior to their first SARS-CoV-2 infection (2: ‘Fully vaccinated’, 1: ‘Partially vaccinated’, 0: ‘Unvaccinated’), as previous studies reported that COVID-19 vaccines reached peak effectiveness at least 14 days after administration.^21^ Those unvaccinated on their first SARS-CoV-2 infection were excluded from the cohort.

### Case-control matching

Within each assessment window suitable control patients were selected, where candidates, not containing an eligible CHD inclusion code, were matched within the following framework: sex, year of birth, ethnicity and general practice, to reduce confounding. No control sample was matched to more than one case. For the postvaccination window, controls were additionally required to have the same vaccination status (partial or full). See further details in online supplemental methods.

### COVID-19 outcomes

Severe COVID-19 outcomes were defined as hospitalisation and death. For each patient’s earliest SARS-CoV-2 infection (baseline date), outcomes of the infection were identified. COVID-19 hospital admissions were identified from secondary care data sets. COVID-19 deaths were defined by a record in the Civil Registration of Death with COVID-19 as a cause of death, or a death record in COVID-19 Hospitalisation in England Surveillance System (online supplemental methods).

### Cardiac inflammation and thrombotic events after vaccination

The incidence rates of inflammatory and thrombotic events (myocarditis, pericarditis, ischaemic stroke, ischaemic heart disease and venous thromboembolic disease) were assessed across the entire time course of the study: from 1 March 2020 to 1 April 2022. Here, cases and controls with no confirmed SARS-CoV-2 infection during the study window were included. Diagnoses of these events were identified from codes present in HES-OP, HES-APC and GDPPR (online supplemental table 1D). Incidence rates were assessed in the 14 days following each patient’s second COVID-19 vaccination (as we observed the highest incidence rate of all complications after this dose in preliminary analyses). Baseline incidence rates were calculated for patients who did not receive a second vaccination during the study window, prior to any COVID-19 infection or first vaccination. Only diagnoses more than 14 days after a previous diagnosis were included, to prevent double counting. Incidence rates were calculated as number of distinct diagnoses per person-year.

### Statistical analysis

Conditional logistic regression was used to compare the odds of a specified COVID-19 outcome (hospitalisation or death) between CHD cases and controls. Separate regression models were fitted for the prevaccination and postvaccination cohorts. The variables used for matching were included as strata in each model, with smoking status included as a covariate. In the postvaccination cohort, the interval between most recent vaccination and first SARS-CoV-2 infection was included as a predictor in each model to examine the reduction in vaccine efficacy over time. Full details of the statistical analysis can be found in the online supplemental material.

This analysis was performed according to a prespecified analysis plan published on GitHub, along with the phenotyping and analysis code (https://github.com/BHFDSC/CCU068_01).

## Results

### Characteristics of prevaccination and postvaccination study cohorts

During the prevaccination window from 1 March 2020 to 8 December 2020, 7805 CHD cases infected with COVID-19 were identified and matched to 27 620 controls. During the postvaccination assessment window from 1 March 2021 to 1 April 2022, 57 550 CHD cases meeting eligibility criteria were identified and matched to 225 160 controls (3.9 controls per case). As specified by the eligibility criteria, included patients were infected with SARS-CoV-2 during the window and vaccinated at least once prior to infection. 92.0% of patients had received two or more vaccinations on SARS-CoV-2 infection. Among patients with a recorded vaccination brand, 52.1% received the BNT162b2 vaccine, 47.5% received ChAdOx1, and 0.4% received mRNA-1273 for both first and second doses. 18% of patients had no identifiable vaccine type.

**Figure 1:**
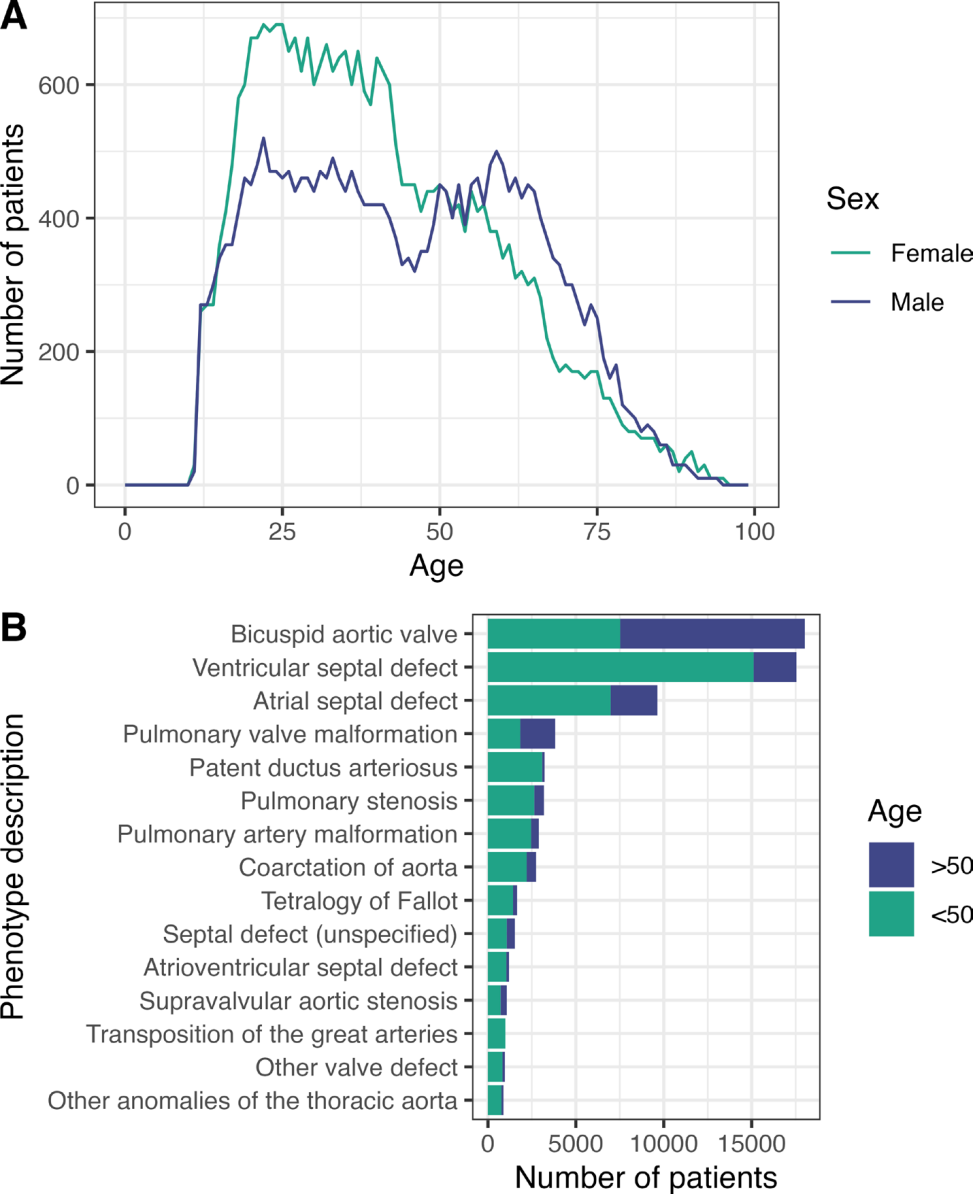
(A) The age distribution of 57 550 vaccinated patients with CHD at the beginning of the postvaccination study period (March 2021), grouped by sex. (**B**). The phenotypical make-up of 57 550 vaccinated patients with CHD. Bars show the percentage of CHD cases with any diagnostic or procedural code for each of the 15 most common CHD phenotypes in the cohort. Each phenotype bar is split to represent the number of over-50s and under-50s with each phenotype. CHD, congenital heart disease.

The characteristics of the study cohorts are described in table 1 and are broadly comparable. The mean age of CHD cases in the postvaccination cohort was 42.4 years. Under-18s were underrepresented in the cohort compared with the general population (7.0% of total cases) as vaccination was only offered to at-risk under-18s in July 2021.^22^ The most frequently observed age was 22 years. While the number of female cases in each age bracket declined with increasing age, male cases displayed a bimodal distribution with peaks at age 20–30 years and 50–60 years (figure 1A), reflecting the threefold higher prevalence of bicuspid aortic valve (BAV) in men. Additionally, the control group had a significantly higher percentage of current smokers than the case group (12.3% vs 9.8%, p<0.0001). The case group had higher rates of comorbid conditions associated with increased COVID-19 risk including obesity, diabetes, heart failure, renal failure and chronic obstructive pulmonary disease (table 2).

**Table 1:** Characteristics of the English prevaccination and postvaccination study cohorts. Variables used to match cases and controls within each cohort are highlighted in bold. ‘Partial’ vaccination status refers to patients who received one vaccination 14 days prior to their first SARS-CoV-2 infection. ‘Full’ vaccination status refers to patients who received two or more vaccinations 14 days prior to their first SARS-CoV-2 infection

	Prevaccination cases	Prevaccination controls	Postvaccination cases	Postvaccination controls
Total number of patients	7805	27 620	57 550	225 160
Mean age, years	44.3	44.1	42.4	42.4
Sex (%)				
Male	47.7	46.9	47.4	47.3
Female	52.3	53.1	52.6	52.7
Ethnicity (%)				
White	90.8	91.4	93.8	94.5
Asian	7.4	7.1	2.6	2.2
Black	0.2	0.2	0.4	0.3
Mixed	0.0	0.0	0.1	0.1
Other	0.3	0.2	0.4	0.3
Unknown	1.3	1.1	2.8	2.6
Vaccination status (%)				
Partial	N/A	N/A	8.0	7.6
Full	N/A	N/A	92.0	92.4
CHD severity (%)				
Mild	66.5	N/A	68.5	N/A
Moderate	29.1	N/A	27.4	N/A
Severe	4.4	N/A	4.1	N/A
Pulmonary hypertension or cyanosis (%)	2.1	N/A	1.6	N/A
Fontan circulation (%)	0.1	N/A	0.1	N/A

CHDcongenital heart disease

**Table 2:** COVID-19 risk factors among the prevaccination and postvaccination study cohorts. χ^2^ test values of p are reported for congenital heart disease case and control groups within each study window

	Prevaccination cases	Prevaccination controls	P value (prevaccination case vs control)	Postvaccination cases	Postvaccination controls	P value (postvaccination case vs control)
Smoking (%)			<0.0001			<0.0001
Current smoker	8.9	10.5		9.8	12.3	
Former smoker	24.0	24.2		23.1	23.1	
Never smoked	59.1	56.7		61.4	57.9	
Unknown	8.0	8.6		5.7	6.7	
Diabetes (%)	14.6	10.2	<0.0001	8.8	6.7	<0.0001
Obesity (%)	30.8	28.4	<0.0001	27.4	25.6	<0.0001
Heart failure (%)	13.3	3.0	<0.0001	8.5	1.4	<0.0001
End-stage renal disease (%)	1.7	0.5	<0.0001	1.0	0.3	<0.0001
COPD (%)	5.8	4.1	<0.0001	3.2	2.2	<0.0001

COPDchronic obstructive pulmonary disease

### Impact of vaccination on excess risks of severe COVID-19 outcomes in patients with CHD

Among unvaccinated people infected with SARS-CoV-2 for the first time during the prevaccination window, patients with CHD faced a significantly increased risk of severe COVID-19 outcomes compared with controls. The rate of hospitalisation with first SARS-CoV-2 infection was 15.8% in unvaccinated CHD cases (n=1230/7805) and 10.0% (n=2720/27 260) in unvaccinated controls without CHD (OR 1.95 (1.77–2.13); p<0.0001). The rate of death from first SARS-CoV-2 infection was 4.6% in CHD cases (n=360) and 3.4% (n=925) in controls (OR=1.49 (1.28–1.73); p<0.0001).

**Figure 2:**
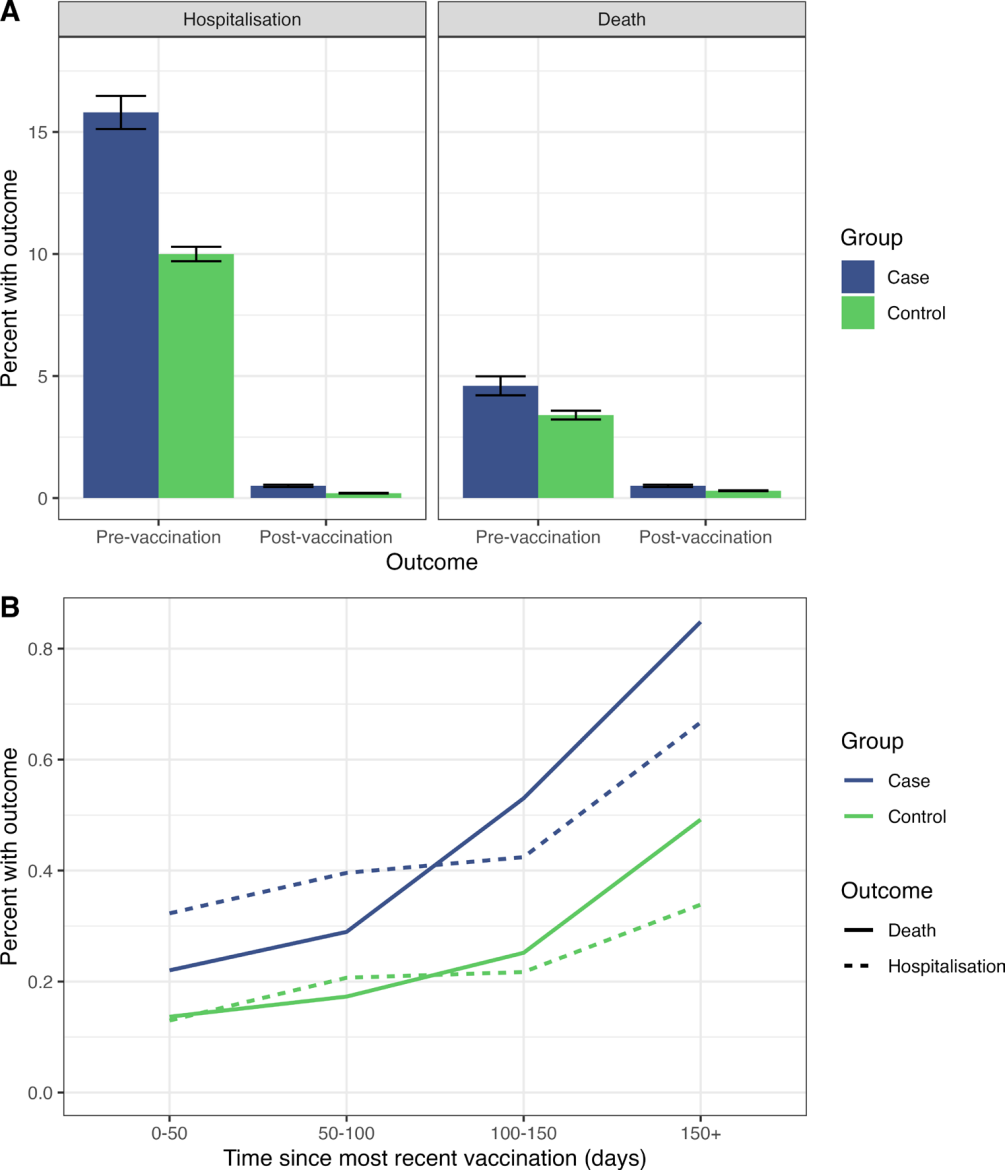
A. The rate of hospitalisation and death from first SARS-CoV-2 infection among 7805 unvaccinated patients with congenital heart disease (CHD) and matched controls and 57 550 vaccinated patients with CHD and matched controls. (**B.**) The rate of hospitalisation and death from first SARS-CoV-2 infection among 57 550 vaccinated patients with CHD and matched controls, grouped by time since the most recent vaccination.

A lower rate of COVID-19-related hospitalisation and death was observed for vaccinated patients with CHD in the postvaccination cohort compared with unvaccinated patients with CHD in the prevaccination cohort. The rate of hospitalisation with first SARS-CoV-2 infection decreased from 15.8% to 0.5%, while the rate of death decreased from 4.6% to 0.5% (figure 2A).

Among vaccinated people infected with SARS-CoV-2 for the first time during the postvaccination window, a significant excess risk of severe COVID-19 remained for patients with CHD compared with controls. The rate of hospitalisation with first SARS-CoV-2 infection was 0.5% (n=260/57 550) in cases of CHD and 0.2% (n=490/225 160) in controls without CHD (OR 2.24 (1.88–2.65); p<0.0001). The rate of death from first SARS-CoV-2 infection was 0.5% (n=265/57 550) in CHD cases and 0.3% (n=560/225 160) in controls without CHD (OR=1.81 (1.54–2.13); p<0.0001).

The risk of hospitalisation from COVID-19 across the entire cohort increased with each additional day since most recent vaccination (OR 1.003 (1.002–1.005); p<0.0001, per day), as did the risk of death (OR 1.005 (1.004–1.007); p<0.0001, per day). The rate of reduction in vaccine efficacy over time was similar between cases and controls (figure 2B).

### CHD complexity and COVID-19 outcomes in vaccinated patients with CHD

The most prevalent conditions in the cohort were BAV (31.3% of cases) and ventricular septal defect (30.5% of cases) (figure 1B). The complexity of CHD was designated mild in 68.5%, moderate in 27.4% and severe in 4.1% of cases. Within the cohort, 1.6% of cases presented with pulmonary hypertension or cyanosis (PH/C), features of advanced physiological stage CHD.

**Figure 3:**
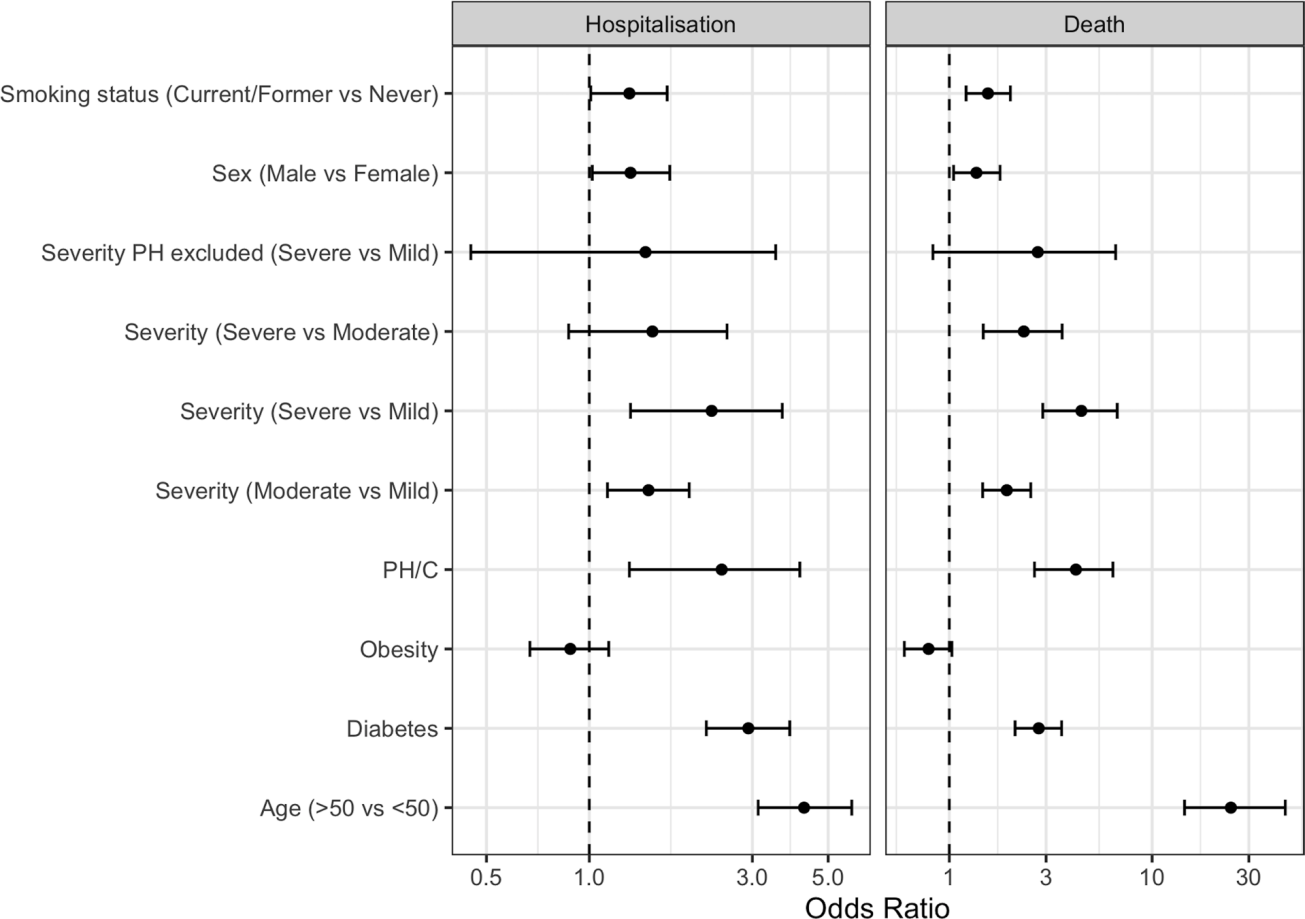
Forest plot of odds ratios (ORs) for hospitalisation and death from first SARS-CoV-2 infection in vaccinated patients with congenital heart disease (CHD). The OR reported is the increase or decrease in odds with the first factor when compared with the second as a reference. The error bars represent 95% CIs for the OR. PH/C, cases with pulmonary hypertension or cyanosis. Values of p<0.05 are highlighted in bold.

Complexity of CHD was associated with the risk of hospitalisation and death from COVID-19. In comparison to mild CHD cases, patients with severe CHD had a significantly increased risk of hospitalisation (OR 2.28 (1.32–3.67); p=0.001) and death (OR 4.48 (2.89–6.73); p<0.0001) from their first SARS-CoV-2 infection (figure 3). Patients with PH/C also had an increased risk of hospitalisation (OR 2.44 (1.31–4.13); p=0.002) and death (OR 4.21 (2.63–6.41); p<0.0001) compared with those without PH/C, similar in magnitude to the excess risk between severe and mild cases. As patients with PH/C were all classified as severe, the comparison between CHD complexities was repeated with patients with PH/C excluded. Among patients without PH/C, there was no longer significant increase in COVID-19 risk associated with severe CHD complexity.

### Other predictors of severe COVID-19 outcomes in vaccinated patients with CHD

The effect of additional variables modifying COVID-19 risk in vaccinated patients with CHD was investigated in a case-only analysis. We considered six additional risk factors for severe COVID-19 outcomes: age, sex, ethnicity, diabetes, obesity and smoking status (figure 3). As expected, the most significant risk factor for severe COVID-19 was age, with those over 50 years old experiencing 24.47 times higher odds of death ((14.45–45.36); p<0.0001) and 4.25 times higher odds of hospitalisation ((3.12–5.86); p<0.0001) than individuals <50 years of age. CHD cases with diabetes also had an increased risk of both hospitalisation (OR 2.92 (2.2–3.86); p<0.0001) and death (OR 2.76 (2.11–3.58); p<0.0001), as did male CHD cases (OR 1.32 (1.02–1.72); p=0.04 and 1.36 (1.05–1.78); p=0.02, respectively), and those with a history of smoking (OR 1.31 (1.02–1.72); p=0.04 and 1.55 (1.21–2.00); p=0.01, respectively). No significant differences in COVID-19 risk were identified between white and non-white patients with CHD, or between obese and non-obese patients with CHD.

### Cardiac inflammation and thrombotic complications after vaccination in patients with CHD

**Figure 4:**
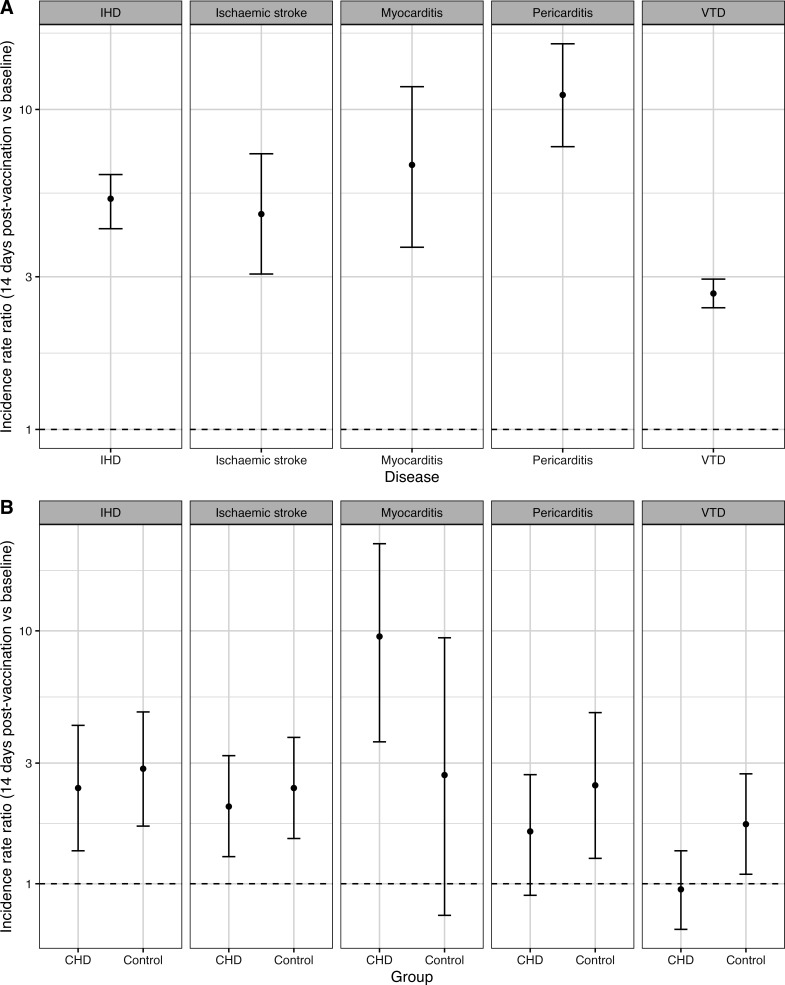
A. Incidence rate ratios for thrombotic and inflammatory events in patients with congenital heart disease (CHD) compared with controls without CHD, prior to SARS-CoV-2 infection or vaccination. (**B**). Incidence rate ratios for thrombotic and inflammatory complications in the 14 days after second COVID-19 vaccination compared with the baseline rate before COVID-19 vaccination or SARS-CoV-2 infection, for patients with CHD and controls. IHD, ischaemic heart disease (new myocardial infarction and onset of angina); VTD, venous thromboembolic disease (deep vein thrombosis, venous thromboembolism and pulmonary embolism).

For CHD cases and controls, the incidence rate of inflammatory and thrombotic events (myocarditis, pericarditis, ischaemic stroke, ischaemic heart disease and venous thromboembolic disease) in the 14 days after receiving a second COVID-19 vaccination of any type was compared with the baseline incidence rate before COVID-19 infection or vaccination. CHD cases had a significantly higher baseline rate of all five conditions (figure 4A). This was particularly pronounced for pericarditis and myocarditis: the baseline incidence rates were, respectively, 11.10 ((7.65–16.04), p<0.0001) and 6.71 ((3.71–11.78), p<0.0001) times higher in CHD cases than in controls (figure 4). However, the ratio between the postvaccination incidence rate and the baseline incidence rate was not significantly higher for patients with CHD than controls for any of the potential associated complications. This indicates that patients with CHD did not have an elevated risk compared with controls after controlling for baseline rates (figure 4B). For patients with CHD, the absolute postvaccination incidence of each complication per patient-year at risk was 0.001 for myocarditis, 0.001 for pericarditis, 0.003 for venous thromboembolic disease, 0.018 for ischaemic stroke and 0.037 for ischaemic heart disease.

## Discussion

Widespread vaccination against COVID-19 has been the primary defence against COVID-19 risks, offering protection against severe illness^23 24^ as well as contributing to broader community immunity. The excess risks of COVID-19 for patients with CHD have previously been described in unvaccinated cohorts.^3^,^5^ We have quantified the reduction in the absolute risk of death and hospitalisation in CHD patients with COVID-19 associated with vaccination in England. However, our data indicate an increased risk of adverse outcomes persists in patients with CHD compared with COVID-19 patients who do not have CHD.

So far there have been very few studies focusing on the effects of vaccination in patients with CHD, with those published only including small, complex adult CHD (ACHD) cohorts. Fusco *et al*^25 26^ studied ACHD patients (n=208 and n=490) from a tertiary centre, but primarily focused on associated adverse effects of vaccination and overall antibody response, rather than vaccine effectiveness. Here, we have analysed a cohort of 57 550 vaccinated CHD patients with COVID-19 in England, with a wide range of anatomical complexity (figure 1), not all of whom would be anticipated to have been receiving care in a tertiary ACHD centre. We find that, in patients with CHD, the rate of COVID-19-related hospitalisation and death fell from 15.8% and 4.6% prevaccination to 0.5% and 0.5%, respectively, following vaccination. Although these risks were significantly reduced, when compared with the matched control group, vaccinated patients with CHD retain additional COVID-19 risk; more than twice the odds of hospitalisation and 81% higher odds of death. Our results therefore emphasise the importance of vaccination in the CHD population and suggest increased vigilance on the part of care providers when dealing with COVID-19 in vaccinated patients with CHD remains appropriate. Additionally, despite the pre-existing baseline risk of cardiac inflammation and thrombotic events in the CHD population (independent of SARS-CoV-2 infection or vaccination), the increase in risk experienced by patients with CHD after vaccination is proportional to that observed in those without CHD receiving vaccination. While this may remain to a degree confounded by comorbidities for which we could not adjust, patients with CHD should be made aware of their higher absolute risk of rare vaccine-associated complications. However, it is crucial to emphasise that these risks are far outweighed by the benefits of protection against severe COVID-19 outcomes.

The overall risk of severe COVID-19 outcomes increases with time following COVID-19 vaccination, while the relative risk between CHD cases and controls is maintained. We found no evidence of differential drop-off in vaccine efficacy between patients with and without CHD. As expected, within the vaccinated CHD cohort, factors such as sex (male) and increased age (>50 years) continued to present increased likelihood of severe outcomes as indicated previously in non-vaccinated patients.^3^ CHD complexity also increased risk, though this excess risk is chiefly determined by accompanying pulmonary vascular disease and/or cyanosis.

This study has some limitations. While our method for distinguishing BAV from early degeneration of a trileaflet valve has previously been shown to be robust,^3 27^ some degree of misclassification is likely to have occurred. Our estimates of the excess risk of CHD among vaccinated patients would therefore most appropriately be regarded as a minimum estimate. While selecting each person’s first documented COVID-19 infection reduced bias due to differences in acquired immunity between the prevaccination and postvaccination cohorts, it was not possible to ensure patients had not had a previous asymptomatic or non-documented infection. We could not adjust our analyses for differences between SARS-CoV-2 strains that emerged during the pandemic due to absence of these data. Regarding the estimation of the incidence of vaccine-associated complications, the number of patients in the vaccinated CHD cohort was too small to enable precise estimates to be calculated for individual vaccine types. Other possible confounders include socioeconomic differences between individuals and complications due to existing comorbidities and/or disease severity that could not be adequately captured by case/control matching. Over the course of the pandemic, clinical practice, hospital admission thresholds and death classifications may have changed, potentially impacting accuracy of the outcome measures used here. The study was initially planned as a two-nation study incorporating Wales. However, the Welsh cohort was removed from the study, being too small for separate statistical analysis.

In summary, we have shown that COVID-19 vaccination is a successful method of mitigating the risk of severe outcomes in patients with CHD. Although reduced, these risks remain higher than non-CHD control patients, particularly among more complex CHD cases. Further work will be required to inform specific interventions by clinical teams managing COVID-19 among higher-risk complex CHD cases, to further mitigate these remaining risks.

## supplementary material

10.1136/heartjnl-2024-324470 online supplemental file 1

10.1136/heartjnl-2024-324470 online supplemental table 1

## Data Availability

Data may be obtained from a third party and are not publicly available.
