# Supplementary material for: The impact of COVID-19 vaccination on patients with congenital heart disease in England: a case-control study
Source: Heart. 2024 Oct 11;110(23):e324470. doi: 10.1136/heartjnl-2024-324470 (PMC11671934; doi:10.1136/heartjnl-2024-324470)
Supplement: online supplemental file 1 [file heartjnl-110-23-s001.pdf]

Supplementary methods

Identification of CHD cases

Patients with specific codes relating to aortic valve disease (Supplementary table 1B) - which could result from a congenitally defective bicuspid aortic valve, or to age-related degeneration of a normal aortic valve - underwent additional filtering steps. Specifically, patients whose first record of aortic valve disease occurred after an age threshold of 65 were excluded. Subsequently, patients whose first record of aortic valve disease occurred after a condition which could damage the aortic valve (Supplementary table 1C) were also excluded. Remaining diagnoses of aortic stenosis, aortic regurgitation, and aortic valve disease were classified as bicuspid aortic valve (1). This process is illustrated in Supplementary Figure 2.

CHD severity classification

European Society of Cardiology guideline incorporate anatomical and physiological aspects of the condition (2). In the event of more than one defect co-existing in the same patient, the classification of the most severe defect was assigned. In line with ESC guidelines, CHD patients with pulmonary hypertension or cyanosis (PH/C) were classified as severe regardless of the specific defect.

According to the ESC guidelines, small, isolated septal defects are mild, while moderate or large septal defects are classified as moderate if unrepaired. The diagnostic codes available in EHRs do not allow determination of the size of a septal defect, therefore, regardless of the presence of OPCS-4 code denoting repair, all isolated septal defects were classified as

mild. Patients with septal defects alongside any other lesion were placed in the moderate category.

CHD patients with pulmonary hypertension or cyanosis were placed in the severe category regardless of the specific defect. The specificity of the available ICD-10 codes did not allow determination of pulmonary hypertension secondary to congenital heart disease. Therefore, patients with codes I27.2 (Other secondary pulmonary hypertension), I27.8 (Other specified pulmonary heart diseases), or I27.9 (Pulmonary heart disease, unspecified) were classified as severe if the code was present alongside a condition with the potential to cause pulmonary hypertension (shunt lesions, left-sided heart disease or pulmonary artery obstructions) (3). Those with the SNOMED code '127063008' or the Read code 'XE1KK', denoting 'Cyanotic congenital heart disease', were also placed in the severe category.

COVID-19 infection and vaccination

Firstly, the first SARS-CoV-2 infection for each patient was identified. Infections were identified in English EHRs by the presence of specific SNOMED codes in the General Practice Extraction Service for Pandemic Planning and Research (GDPPR) (840539006, 1119302008, or 1240581000000104), positive COVID-19 PCR test results in the Second Generation Surveillance System (SGSS), hospital admissions recorded in COVID-19 Hospitalisations in England Surveillance System (CHESS), or ICD-10 codes in Hospital Episode Statistics - Admitted Patient Care (HES-APC) denoting COVID-19 disease (U07.1 or U07.2). From these records of SARS-CoV-2 infection, the earliest for each patient was selected.

For the post-vaccination time window only, the vaccination status of each person upon their first SARS-CoV-2 infection was identified. Records of vaccination were identified from the English Vaccination Status database, as well as from SNOMED (1156257001, 1324681000000101 or 1324691000000104) codes found in the primary care datasets. Codes relating to vaccine type are in Supplementary table 1F.

Case-control matching

Cases were matched to up to four unique controls (4, 5). To enable matching, information for patients with conflicting records for sex, ethnicity, or GP practice had to be resolved. Therefore, the person's most frequently recorded response was chosen in cases where conflicting records were present, and in the event of a tie, the most recent of the tied responses was chosen. Cases for which controls could not be identified underwent a second round of matching, with the date of birth criterion relaxed; cases could be matched to controls with a date of birth within four years of their own. Each control sample was only used once. Upon matching to a case sample, a control sample was removed from further consideration as a control for a different case, or as another control for the same case sample.

COVID-19 outcomes

Severe COVID-19 outcomes were defined as hospitalisation and death. For each patient's earliest Sars-CoV-2 infection (baseline date), the outcome of the infection was identified. COVID-19 hospital admissions were identified from secondary care datasets. COVID-19 deaths were defined by a record in the Civil Registration of Death (England) database with

COVID-19 as a cause of death, or a record in COVID-19 Hospitalisation in England Surveillance System (CHESS) with the outcome stated as 'Death'.

Hospital admissions more than 20 days after a SARS-CoV-2 infection were excluded, to remove hospital admissions relating to a subsequent infection. Deaths more than 28 days after a first SARS-CoV-2 infection were excluded, in line with the measure used by the UKHSA 'to ensure the measure only reflected deaths during the acute phase of infection'(6).

Identifying COVID-19 risk factors

Additional COVID-19 risk factors, including smoking status, obesity, diabetes, end-stage renal disease, chronic obstructive pulmonary disease, and heart failure, were identified for each congenital heart disease case and control from primary care records. The SNOMED codes for these risk factors are detailed in Supplementary Table 1E. For smoking status and obesity, the most recent record of smoking behaviour or BMI was used to determine the current status of each individual.

Statistical analysis

We used Chi-Square tests to compare categorical variables (smoking, diabetes, obesity, heart failure, end-stage renal disease and COPD) between case and controls groups in both the post- and pre-vaccination cohorts (table 2).

Conditional logistic regression was used to compare the odds of a specified COVID-19 outcome (hospitalisation or death) between CHD cases and controls. Separate regression models were fitted for the pre-vaccination and post-vaccination cohorts. The variables used

for matching were included as strata in each model, with smoking status included as a covariate. In the post-vaccination cohort, the interval between most recent vaccination and first SARS-CoV-2 infection was included as a predictor in each model to examine the reduction in vaccine efficacy over time.

A case-only analysis was performed to investigate the influence of age, ethnicity, sex, CHD severity, and presence of pulmonary hypertension and/or cyanosis (PH/C) on COVID-19 outcomes in the post-vaccination cohort. The age variable was dichotomised using a threshold of age 50 years, and ethnicities were categorised into 'White', 'Non-white', and 'Unknown'. We did not attempt to precisely quantify the risk associated with age; instead, this was principally to demonstrate that, despite vaccination, known risk factors persist in CHD patients. Multivariable logistic regression was used to estimate the effect of each variable on the odds of an outcome (hospitalisation/death) from a patient's first SARS-CoV-2 infection. Then, patients with PH/C were removed from the analysis and odds ratios recalculated to estimate the effect of severity independently of PH/C.

The incidence rate ratios of secondary events after COVID-19 vaccination compared to baseline were estimated using negative binomial regression, adjusting for age and sex. Subgroup analyses were conducted between CHD cases and controls, and tests for interaction were carried out using ANOVA.

Patients with an invalid or missing date of birth were excluded, and CHD cases with an unknown ethnicity were matched to controls with an unknown ethnicity. In the conditional

logistic regression, a separate category was created for individuals with missing smoking status, denoted as ‘unknown’ which accounted for less than 10% of observations in both cohorts.

Supplementary results

CHD subgroup analysis

CHD patients were split into those with bicuspid aortic valve (BAV) and those with non-BAV CHD and compared in terms of their COVID-19 outcomes in the pre- and post-vaccination windows (supplementary table 3). Pre-vaccination, patients with BAV had worse COVID-19 outcomes compared to their matched controls than did non-BAV CHD patients (p=5.00e-4 and p=3.80e-3 for hospitalisation and death, respectively). Post-vaccination, BAV and non-BAV CHD patients had no significant difference from each other (p=2.30e-1 and p=2.70e-1 for hospitalisation and death respectively). Patients with BAV are often excluded from cohort studies of CHD, predominantly due to their mild defect and differing age-distribution compared to complex CHD. However, BAV is associated with various additional cardiovascular complications, including aortic stenosis and dilation, as well as comorbidities like hypertension, that can exacerbate the effects of severe systemic illness such as COVID-19. These findings highlight the importance of including BAV to capture real-world CHD patients, as severe COVID-19 outcomes are evident.

Supplementary tables and figures

**CHD subtypes included in analysis**
anomalous origin of the right coronary artery from pulmonary artery
anomalous pulmonary venous connection
aortic regurgitation (in the context of BAV)
aortic stenosis (in the context of BAV)
aortic valve atresia/aplasia
aortic valve disease (in the context of BAV)
atrial septal defect
atrioventricular septal defect
bicuspid aortic valve (BAV)
coarctation of aorta
coarctation of pulmonary artery
common arterial trunk
common atrioventricular valve
congenital mitral atresia
congenital mitral regurgitation
congenital mitral stenosis
congenital mitral valve disease
congenital valve defect (not otherwise specified)
double inlet left ventricle
double inlet right ventricle
double inlet ventricle
double outlet left ventricle
double outlet right ventricle
double outlet ventricle
ebstein anomaly
general CHD classification (not otherwise specified)
hypoplastic left heart
hypoplastic right heart
interrupted aortic arch
left atrioventricular valve
left ventricular outflow obstruction
other anomalies of the thoracic aorta
patent ductus arteriosus
pulmonary artery atresia
pulmonary artery malformation

pulmonary stenosis
pulmonary valve agenesis/atresia
pulmonary valve malformation
pulmonary vein malformation
right atrioventricular valve
right ventricular outflow obstruction
septal defect (unspecified)
single ventricle
sinus of valsalva
subaortic stenosis
supravalvular aortic stenosis
tetralogy of fallot
total anomalous pulmonary venous connection
transposition of the great arteries
tricuspid atresia
unicuspid aortic valve
ventricular septal defect

Supplementary table 2. A list of the CHD subtypes included in the analysis.

Period	Group	Outcome	n	% with outcome	OR (vs control)	p-value	p.value (BAV vs non-BAV CHD)
Pre-vaccination	BAV	Hospitalisation	2044	26.8	2.13 [2.02-2.66]	5.56E-33	5.00E-04
Pre-vaccination	non-BAV CHD	Hospitalisation	5761	11.8	1.68 [1.48-1.90]	3.68E-16	
Pre-vaccination	BAV	Death	2044	8.51	1.90 [1.53-2.38]	1.21E-08	3.80E-03
Pre-vaccination	non-BAV CHD	Death	5761	3.26	1.27 [1.03-1.56]	7.62E-05	
Post-vaccination	BAV	Hospitalisation	14579	0.79	2.51 [1.95-3.26]	2.28E-12	2.30E-01
Post-vaccination	non-BAV CHD	Hospitalisation	42975	0.34	2.05 [1.64-2.58]	4.82E-10	
Post-vaccination	BAV	Death	14579	0.82	2.01 [1.57-2.56]	2.11E-08	2.70E-01
Post-vaccination	non-BAV CHD	Death	42975	0.34	1.68 [1.35-2.07]	2.38E-06	

Supplementary table 3. A comparison of COVID-19-related hospitalisation and death in patients with bicuspid aortic valve (BAV) and non-BAV CHD conditions.

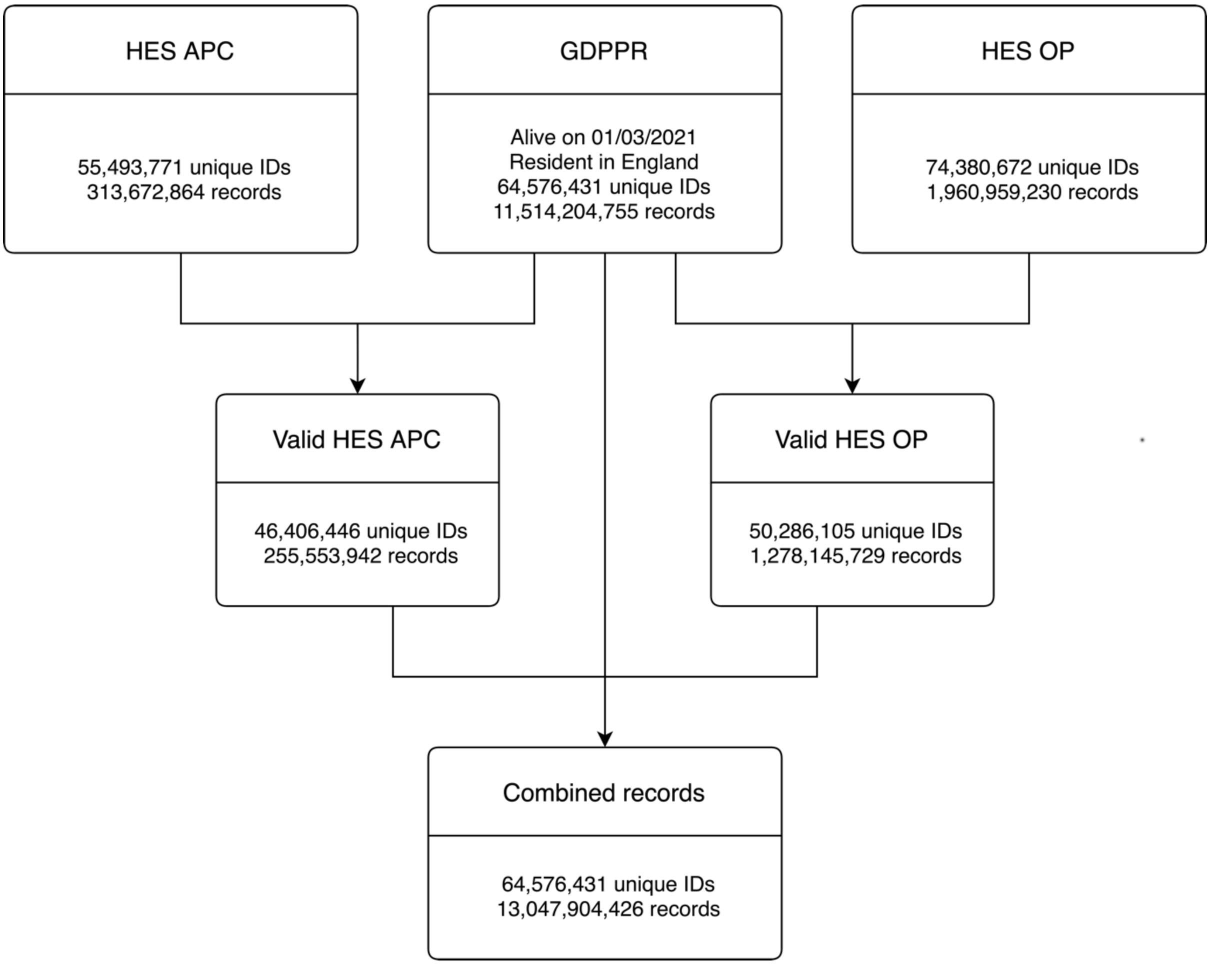

Supplementary Figure 1. Flowchart showing linkage of English Electronic Health Records to select the post-vaccination study cohort. 1) Records from HES OP (Outpatient Hospital Episode Statistics) and HES APC (Admitted Patient Care Hospital Episode Statistics) were kept if they had an NHS number matching one in the validated GDPPR (General Practice Extraction Service Data for Pandemic Planning and Research) records. 2) Validated records from GDPPR, HES OP and HES APC were combined into a single table.

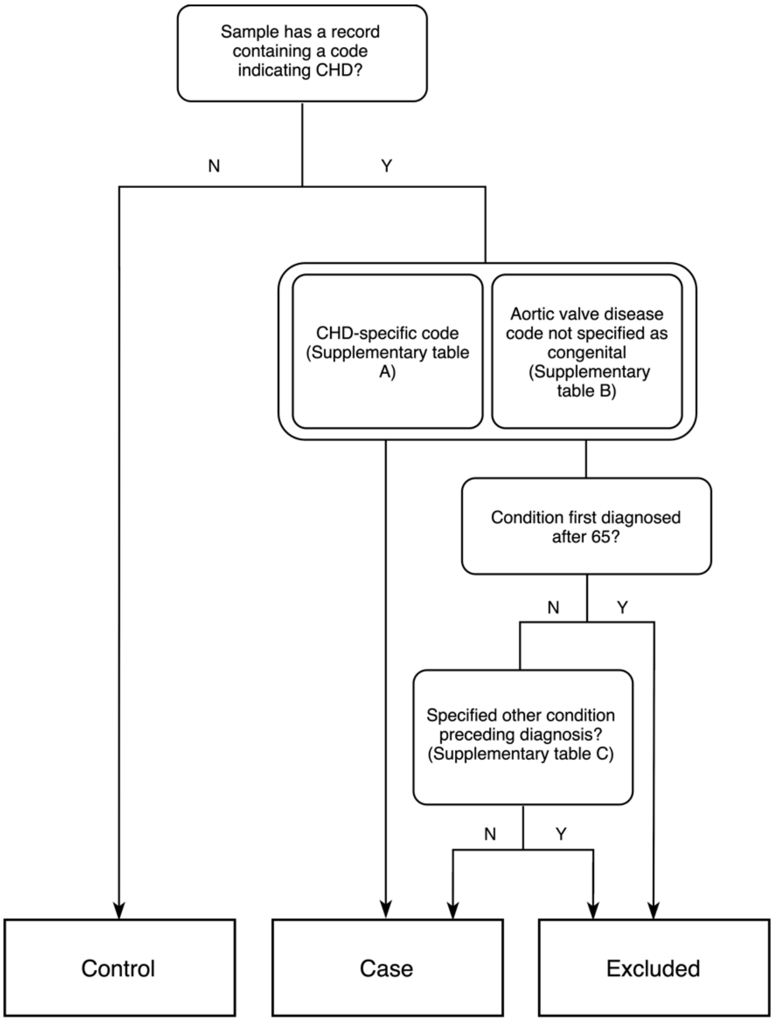

Supplementary Figure 2. Flowchart showing the process of classifying patients into cases and controls based on diagnosis and procedural codes found in electronic health records. Codes used for filtering samples include ICD-10 diagnosis codes, OPCS-4 procedural codes and SNOMED codes. The specific codes can be found in Supplementary Table 1 (A, B, and C).

References

1. Roberts WC, Ko JM. Frequency by decades of unicuspid, bicuspid, and tricuspid aortic valves in adults having isolated aortic valve replacement for aortic stenosis, with or without associated aortic regurgitation. Circulation. 2005;111(7):920-5.
2. Baumgartner H, De Backer J, Babu-Narayan SV, Budts W, Chessa M, Diller GP, et al. 2020 ESC Guidelines for the management of adult congenital heart disease. Eur Heart J. 2021;42(6):563-645.
3. Pascall E, Tulloh RM. Pulmonary hypertension in congenital heart disease. Future Cardiol. 2018;14(4):343-53.
4. Rothman KJ. Modern Epidemiology1986.

5. Hennekens CH, Buring, J.E. Epidemiology in Medicine1987.
6. Seghezzo G, Allen H, Griffiths C, Pooley J, Beardsmore L, Caul S, et al. Comparison of two COVID-19 mortality measures used during the pandemic response in England. Int J Epidemiol. 2023.
